# Novel germline variants of *CDKN1B* and *CDKN2C* identified during screening for familial primary hyperparathyroidism

**DOI:** 10.1007/s40618-022-01948-7

**Published:** 2022-11-05

**Authors:** I. Mazarico-Altisent, I. Capel, N. Baena, M. R. Bella-Cueto, S. Barcons, X. Guirao, L. Albert, A. Cano, R. Pareja, A. Caixàs, M. Rigla

**Affiliations:** 1 Endocrinology and Nutrition Department, Parc Taulí University Hospital, Institut d’Investigació i Innovació Parc Taulí (I3PT), Medicine Department, Universitat Autònoma de Barcelona, Parc Taulí 1, 08208 Sabadell, Barcelona Spain; 2 Genetic Department, Parc Taulí University Hospital, Institut d’Investigació i Innovació Parc Taulí (I3PT), Medicine Department, Universitat Autònoma de Barcelona, Parc Taulí 1, 08208 Sabadell, Barcelona Spain; 3 Pathology Department, Parc Taulí University Hospital, Institut d’Investigació i Innovació Parc Taulí (I3PT), Medicine Department, Universitat Autònoma de Barcelona, Parc Taulí 1, 08208 Sabadell, Barcelona Spain; 4 Surgery Department, Parc Taulí University Hospital, Institut d’Investigació i Innovació Parc Taulí (I3PT), Medicine Department, Universitat Autònoma de Barcelona, Parc Taulí 1, 08208 Sabadell, Barcelona Spain

**Keywords:** Parathyroid, Primary hyperparathyroidism, Multiple endocrine neoplasia type 1, Multiple endocrine neoplasia type 4, Cyclin dependent kinase inhibitors

## Abstract

**Purpose:**

*CDKN1B* mutations were established as a cause of multiple endocrine neoplasia 4 (MEN4) syndrome in patients with MEN1 phenotype without a mutation in the *MEN1* gene. In addition, variants in other cyclin-dependent kinase inhibitors (*CDKIs*) were found in some MEN1-like cases without the *MEN1* mutation. We aimed to describe novel germline mutations of these genes in patients with primary hyperparathyroidism (PHPT).

**Methods:**

During genetic screening for familial hyperparathyroidism, three novel *CDKIs* germline mutations in three unrelated cases between January 2019 and November 2021 were identified. In this report, we describe clinical features, DNA sequence analysis, and familial segregation studies based on these patients and their relatives. Genome-wide DNA study of loss of heterozygosity (LOH), copy number variation (CNV), and p27/kip immunohistochemistry was performed on tumour samples.

**Results:**

DNA screening was performed for atypical parathyroid adenomas in cases 1 and 2 and for cystic parathyroid adenoma and young age at diagnosis of PHPT in case 3. Genetic analysis identified likely pathogenic variants of *CDKN1B* in cases 1 and 2 and a variant of the uncertain significance of *CDKN2C*, with uniparental disomy in the tumour sample, in case 3. Neoplasm screening of probands showed other non-endocrine tumours in case 1 (colon adenoma with dysplasia and atypical lipomas) and case 2 (aberrant T-cell population) and a non-functional pituitary adenoma in case 3.

**Conclusion:**

Germline mutations in *CDKIs* should be included in gene panels for genetic testing of primary hyperparathyroidism. New germline variants here described can be added to the current knowledge.

**Supplementary Information:**

The online version contains supplementary material available at 10.1007/s40618-022-01948-7.

## Introduction

Multiple endocrine neoplasia type 1 (MEN1) is a syndrome characterized by the pituitary, parathyroid and entero-pancreatic neuroendocrine tumours (NETs). Primary hyperparathyroidism (PHPT) affects 95% of MEN1 patients and is its most common clinical feature [1, 2]. This syndrome is caused by loss-of-function variants of the *MEN1* tumour-suppressor gene (11q13), which encodes menin, a protein responsible for genome stability, transcription, and cell division [3–6]. Germline mutations of *MEN1* gene have been found in around 85% to 90% of clinically suspected MEN1 patients [7]. Thus, in a meaningful number of patients with MEN1-like phenotype, around 10%, germinal *MEN1* mutations are not detected [4, 8–10]. This lack of detection could possibly be explained by genetic abnormalities in non-coding regions or whole-gene deletion of *MEN1*, however, large deletions of *MEN1* appear to be uncommon [5, 11–14]. Therefore, other predisposing genes, such as cyclin-dependent kinase inhibitors (*CDKIs*), have been suggested by different authors to play a role in patients with MEN1-like phenotype without the *MEN1* mutation [12, 15, 16] since it is known that menin affects gene transcription of some cell cycle regulators, such as *CDKN1B*/p27 and *CDKN2C*/p18 [17]. In particular, *CDKN1B* germline pathogenic variants have been reported in some patients with MEN1-like phenotype without a *MEN1* gene mutation, a condition termed MEN4 syndrome (OMIM 610755) [12, 14, 18–20].

PHPT is the most common clinical feature of both MEN1 and MEN4 syndromes. It is known that 10% of cases of PHPT are hereditary due to germline mutations of different genes, a condition called familial PHPT (FHPT) [1]. FHPT involves syndromic forms, such as multiple endocrine neoplasia types 1 to 4 and hyperparathyroidism-jaw tumour syndrome. Besides, FHPT may present as non-syndromic PHPT, which includes familial hypercalcemia hypocalciuric forms, neonatal severe hyperparathyroidism, and others, that are associated with several genes and are all classified as familial isolated hyperparathyroidism [21]. Until now, a consensual systematized genetic diagnosis for FHPT has not been implemented due to a lack of firm evidence about which individuals have to be tested and which mutations should be determined. Even so, most authors agree on the recommendation of testing for germline mutations in individuals with PHPT plus some specific situations: (1) PHPT occurring before the age of 45 years, (2) recurrent or persistent PHPT, (3) multi-gland disease (MGD), (4) atypical parathyroid adenoma (APA), (5) parathyroid carcinoma, (6) existence of other tumours related to syndromic PHPT, and/or (7) being a first-degree relative of a known mutation carrier or having a first-degree relative with PHPT [22, 23]. Germline mutation testing for possible FHPT may include *MEN1*, *CASR*, *CDC73, CDKN1A, CDKN1B, CDKN2B, CDKN2C, RET, PTH, GNA11, AP2S1, GCM2*, and *AIP* genes according to recent data [1, 23, 24].

Minimal information concerning MEN1-like patients due to *CDKIs* germline mutations have been reported to date, and this lack of reporting suggests that this type of FHPT probably remains underdiagnosed. This report describes three individuals with a history of PHPT who were evaluated based on a suspicion of FHPT and carried novel germline variants of *CDKIs* genes.

## Material and methods

### Phenotype

We re-evaluated data from three patients with a history of PHPT in whom we performed genetic screening for suspicion of FHPT. Clinical information about probands and their first-degree relatives (including clinical features, biochemical results, and radiological findings) was obtained from medical records and routine visits in our hospital (Endocrinology and Nutrition Department). Table 1 presents the clinical findings.

**Table 1:** Clinical characteristics of patients

	Mutation	LOH/UPD	PHPT history			Screening of related tumors after genetic diagnosis			Additional Medical History of Endocrine or Cancer Disorders (or remarkable)
			Ca max (mmol/L)	PTH max (pmol/L)	Histology diagnosis	Pituitary MRI	CT thorax and abdomen	Abnormal lab tests	
Case 1	*CDKN1B* c.280_281delinsG, p.(Pro94Alafs*25)	No	3.39	4,950	APA	Lack of neurohypophyseal bright signal	Enlarged right adrenal gland Bilateral renal cysts	CgA: 493.9 μg/L	Colon tubule-villous adenoma with low-grade dysplasia Atypical lipomas Obesity T2DM Primary hypothyroidism
Case 2	*CDKN1B* c.169C>T, p.(Gln57*)	No	3.37	33,963	MGD: 3 glands parathyroid hyperplasia and 1 APA	Normal	No tumours	CgA: 1,947.1 μg/L Gastrin: 132.6 pmol/L Prl: 6,127.2 pmol/L FSH: 106 UI/L LH: 79.4 UI/L T(t): 4.23 nmol/L T(f): 0.01 nmol/L IGF1: 5.85 nmol/L	Subclinical hypothyroidism Obesity Aberrant T-cell population Chronic idiopathic axonal polyneuropathy
Case 3	*CDKN2C* c.319T>G, p.(Leu107Val)	Yes	2.84	846	MGD: 1 cystic adenoma and 1 parathyroid adenoma	Nonfunctioning cystic microadenoma	No tumours	CgA 110.3 μg/L Prl 1,358.6 pmol/L UFC 225.5 pg/24 h	Uterine leiomyomas Bilateral ovarian cysts

LOH: loss of heterozygosity; UPD: uniparental disomy; RV: reference value; Ca max: higher blood calcium levels before parathyroid surgery (RV: 2.1–2.55); PTH max: serum higher parathyroid hormone before parathyroid surgery (RV: 95–618); APA: atypical parathyroid adenoma; MGD: multiglandular disease; CgA: chromogranin A (RV < 100); T2DM: type 2 diabetes mellitus; Prl: Prolactin (RV 212.6–1,034.5); Gastrin RV 6.2–54.8; UFC: Urinary free cortisol (RV 36–137), FSH: follicle-stimulating hormone (RV: 1.5–12.4); LH: luteinizing hormone (RV: 1.7–8.6); T(t): total testosterone (RV: 6.69–25.6); T(f): free testosterone (RV: 0.02–0.08); IGF1: insulin-like growth factor 1 (RV: 7.54–26.52); MRI: magnetic resonance imaging; CT: computed tomography

### Gene panel sequencing and variant interpretation

DNA was extracted from blood and other tissues samples using the Gentra Puregene DNA reagent (Qiagen, Valencia, CA). The customized gene panel of FHPT screening in our centre includes sequence analysis and copy number variation (CNV) analysis of the following genes: *AIP*, *AIRE*, *AP2S1*, *CASR*, *CDC73*, *CDKN1A*, *CDKN1B*, *CDKN2B*, *CDKN2C*, *GCM2*, *GNA11*, *MEN1*, *PTH*, *RET*, and *TRPV6*.

In the analysis, the mean sequencing depth was > 150 times, and > 99% of target nucleotides were covered with > 20 × sequencing depth for all assays. The target nucleotides included all protein-coding exons of the genes on the panels in addition to 20 base pairs (bp) inside each intron/exon boundary. The panel was also customized by adding oligonucleotides that targeted deep intronic variants (≥ 20 bp from the intron/exon boundary) and non-coding variants (promoter region, 5′ or 3′ untranslated regions [UTR]), which have been reported as disease causing in association with hyperparathyroidism. Sequence reads of each sample were mapped to the human reference genome (GRCh37/hg19). Burrows–Wheeler Aligner (BWA–MEM) software was used for reading alignment. Duplicate read marking, local realignment around indels, base quality score recalibration, and variant calling were performed using genomic analysis toolkit (GATK) algorithms (Sentieon) for nDNA. Variant data were annotated using a collection of tools (VcfAnno and VEP) with a variety of public variant databases, including but not limited to Genome Aggregation Database control population cohorts (gnomAD), ClinVar, and Human Gene Mutation Database (HGMD). Duplicate read marking, local realignment around indels, base quality score recalibration and variant calling were performed using GATK algorithms [25].

The sequence variant analysis pipeline was validated in a Clinical Laboratory Improvement Amendments (CLIA) and College of American Pathologists (CAP) accredited Blueprint Genetics diagnostic laboratory. The series of selected quality criteria included a variant call quality score, variant genomic location, sequence content, and integrative genomics viewer visual analysis. This algorithm was established based on the outcome of an internal validation performed in the CLIA and CAP-accredited Blueprint Genetics diagnostic laboratory.

CNV analysis was performed bioinformatically from next-generation sequencing (NGS) data using a bioinformatic pipeline; one component is a CNV kit and another one involves in-house developed proprietary technology. CNVs were confirmed using digital polymerase chain reaction (PCR). The CNV analysis pipeline was validated in the CLIA and CAP-accredited Blueprint Genetics diagnostic laboratory.

Sanger sequencing of the candidate variants was performed on the patient and the family member samples to confirm the presence of the variant and the pattern of inheritance. Variants were classified following the American College of Medical Genetics and Genomics and the Association for Molecular Pathology (ACMG/AMP) guidelines [26].

### Study of loss of heterozygosity (LOH) and CNV in tumour

Tumour tissue samples from formalin-fixed, paraffin-embedded (FFPE) were used for this analysis. DNA of tumour samples from patients who had undergone parathyroidectomy was extracted using Qiagen DNA extraction kit (QIAGEN, Valencia CA). Genome-wide DNA study of LOH and CNV was performed using OncoScan CNV Assay (Thermo Fisher Scientific, Inc., Waltham, MA, USA). This assay is designed to cover the entire genome with higher resolution in well-known cancer drivers (900 cancer genes). Data analysis was done using the software Chromosome Analysis Suite 4.3 (ChAS 4.3) for CNV and LOH detection.

### Immunohistochemistry

Immunohistochemical study with antip27/kip1 antibody (clone SX53G8, Ventana) was performed on slides obtained from formalin-fixed paraffin-embedded parathyroidectomy tissues using the Ventana Benchmark immunostaining system (Ventana Medical System, Tucson, USA). Endothelial cells were used as positive controls. Immunohistochemistry for p18 was not performed due to the lack of a commercially available monoclonal antip18 antibody.

## Results

### Case 1

#### Medical history and clinical presentation

A 47-year-old man was referred to our department for a cervical node. In his personal history, he presented obesity, primary hypothyroidism, and obstructive sleep apnoea syndrome. In addition, he had undergone surgery for two atypical lipomas and had an endoscopic polypectomy of a tubule-villous adenoma with low-grade dysplasia. He had no family history of parathyroid disorders.

On examination, he had no obvious neck masses or goiter. A neck ultrasound scan (US) showed a homogeneously hypoechoic nodule of 3 cm posterior to the thyroid gland, suggesting a parathyroid lesion. Blood tests revealed severe hypercalcemia (3.39 mmol/L; reference value [RV] 2.1–2.55), hypophosphatemia (0.58 mmol/L, RV 0.87–1.45), and elevated parathyroid hormone (PTH) levels (4950 pmol/L, RV 95–618), consistent with PHPT (Table 1). No clinical history of fractures or nephrolithiasis was present. Treatment was initiated with cinacalcet and oral hydration, and he underwent surgical removal of a solitary abnormal parathyroid gland at the age of 48. Intra-operative PTH (ioPTH) monitoring confirmed the excision of the adenoma as the PTH dropped from 4950 pmol/L (pre-excision) to 618 pmol/L (30′ after excision). Histological study evidenced a nodule (size: 4.5 × 3.5 × 2.5 cm; weigh: 15.9 g) with histopathological characteristics consistent with an atypical parathyroid adenoma (APA), such as fibrous bands associated with haemosiderin deposition and mitotic activity (2 mitotic events/10 high-power fields). Proliferation index (Ki67) was 3%.

After surgery, serum calcium nadir was 2.2 mmol/L, and during the next 10 years, it oscillated between 2.07 and 2.56 mmol/L. Ionic serum calcium remained within normal limits until now.

Given the atypical features of adenoma, genetic testing was performed. Sequence analysis using the panel described above identified a heterozygous *frameshift* likely pathogenic variant in *CDKN1B* (NM 004064.4) c.280_281delinsG, p.(Pro94Alafs*25).

#### Germline genetic analysis and LOH/CNV study in tumour

This variant deletes two base pairs, inserts one base pair in exon 1 (of three total exons) and generates a *frameshift*, leading to a premature stop codon at position 25 in a new reading frame. This setup is predicted to lead to loss of normal protein function, either through protein truncation or nonsense-mediated mRNA decay. This variant is absent in the gnomAD. To our knowledge, this variant has not been described in the medical literature or reported in disease-related variation databases, such as ClinVar or HGMD. Loss of *CDKN1B* function is an established disease mechanism, and other truncating variants in the gene have been described in patients with phenotypes consistent with *CDKN1B*-related disease, which is inherited in an autosomal dominant (AD) manner. This *CDKN1B* variant is classified as likely pathogenic based on the established association between the gene and the patient’s phenotype, the variant’s absence in control populations, and the variant type (*frameshift*). The genetic analysis of tumour DNA did not show LOH or CNV.

#### p27/kip1 immunohistochemistry

**Fig. 1:**
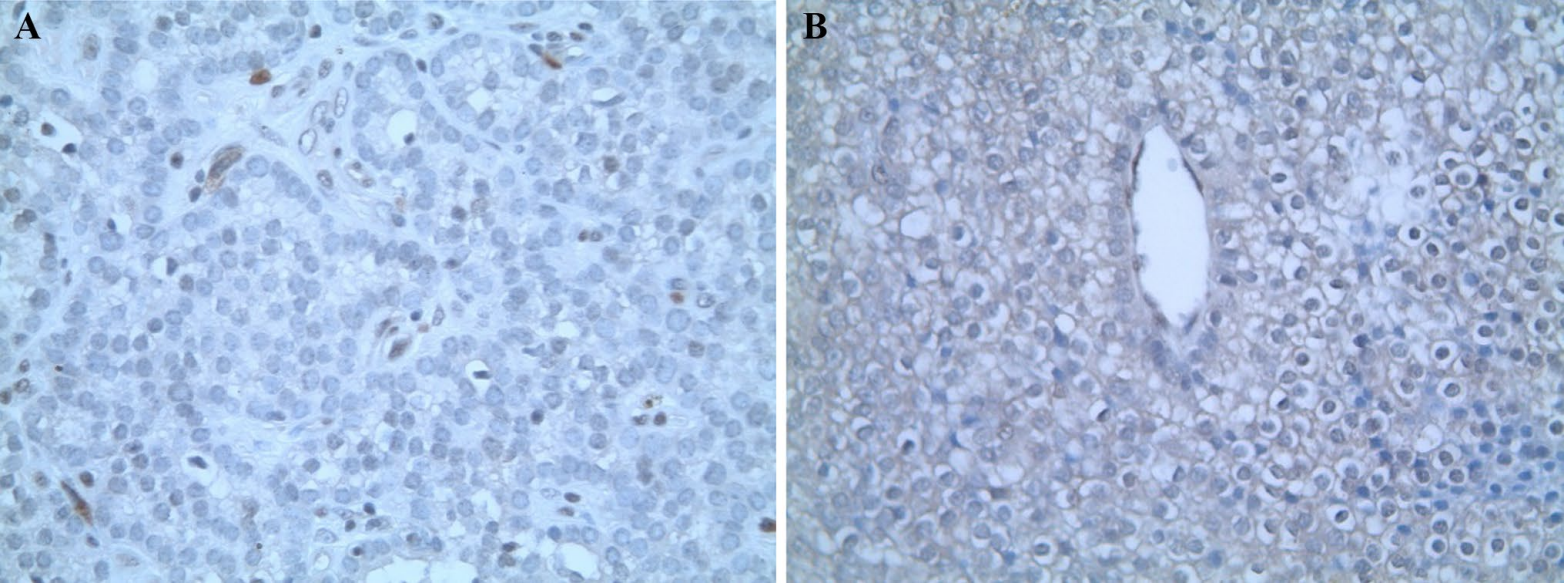
Immunostaining with p27/kip1 of parathyroid adenomas from cases 1 (**a**) and 2 (**b**). Absence of nuclear expression in adenoma cells. × 400

Immunohistochemical analysis of the excised parathyroid adenoma demonstrated loss of nuclear expression of p27/kip1 in neoplastic cells. This result is consistent with a lower expression of the tumour suppressor protein due to the described pathogenic variant (Fig. 1a).

#### Clinical evolution after the genetic result

Specific guidelines for MEN4 management are lacking as few cases have been reported until now, however, most authors recommend following MEN1 guidelines in these cases [7, 27]. Thus, we performed a complete radiological and serological study (Table 1). Lab tests did not show alterations in pituitary hormones, and pituitary magnetic resonance imaging (MRI) manifested a lack of a neurohypohyseal bright signal, which was attributed to a normal physiological occurrence in the absence of any clinical suspicion [28]. A computed tomography (CT) of the chest and abdomen indicated enlarged right adrenal gland and bilateral renal cysts without clinical significance. Urinary free cortisol was normal. Screening for asymptomatic NETs showed a non-specific mild elevated chromogranin A ([CgA] 493.9 μg/L, RV < 100) that has almost normalized over time.

#### Family screening after genetic results

**Fig. 2:**
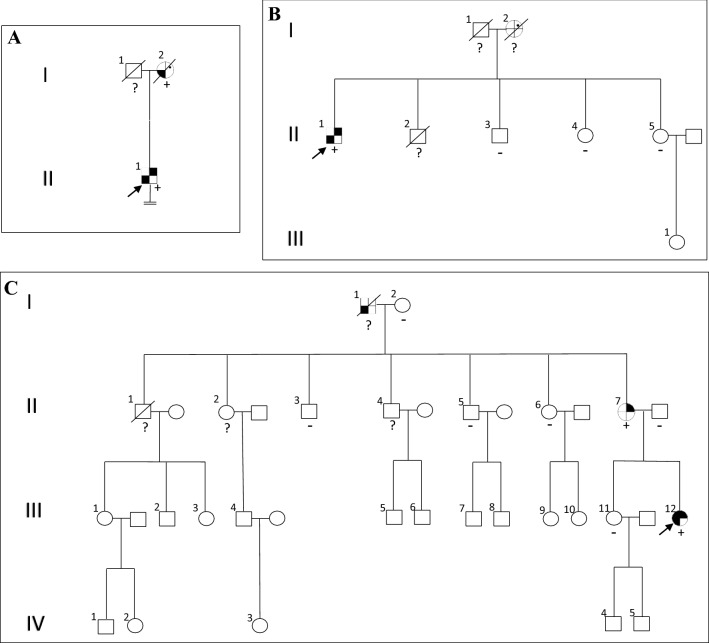
Pedigreeandtumors of probands (withconfirmatedmutation) andtheirrelatives. **a** Case 1, variant c.280_281delinsG, p.(Pro94Alafs*25) CDKN1B; **b** case 2, variant c.169C > T, p.(Gln57*) CDKN1B; **c** case 3, variant c.319 T > G, p.(Leu107Val) CDKN2C. Generation number is indicated with roman numerals. Arrows indicate index case; circle, female; square, male; double line below descent line, no offspring; empty symbol, unaffected family member; slashed symbol, deceased; + , mutation positive; −, mutation negative; ?, no mutation test performed; filled upper right quadrant, PHPT; filled upper rightspot, history of nephrolithiasis, renal colics or hypercalcemia; filled upper left quadrant, pituitary adenoma; filled lower right quadrant, neuroendocrine tumor; filled lower left quadrant, tumor unspecified

A complete family history was taken after the genetic diagnosis was obtained. No first-degree relatives were still alive. Even so, we were able to analyse a DNA sample from a surgical specimen from the mother (I.2, Fig. 2a), who had died years before from a colon neoplasia. In addition, she presented hypercalcemia on some occasions, which had not been evaluated. She carried the same variant in the *CDKN1B* gene as her son.

### Case 2

#### Medical history and clinical presentation

We report a case of a 57-year-old man with a history of chronic kidney disease (CKD) due to interstitial nephropathy diagnosed at the age of 28, which progressed to end-stage renal disease (ESRD) and was treated with haemodialysis since he was 43. As additional significant medical history, he was diagnosed with chronic idiopathic axonal polyneuropathy, aberrant T-cell population at the age of 46, obesity, and subclinical hypothyroidism. As the only family history of interest, his mother had had renal colic. He was diagnosed with tertiary hyperparathyroidism six years after presenting with CKD. Lab tests showed corrected calcium of 3.37 mmol/L and PTH of 33,963 pmol/L. A bone mineral density (BMD) scan was not obtained. He had a previous history of symptomatic kidney stones and complicated urinary infections. Thus, the possibility of having a PHPT prior to CKD could not be excluded. Neck US revealed a hypoechoic nodule (1.3 cm in size) posterior to the thyroid gland. Conversely, scintigraphy study with technetium 99 m sestamibi (TcMIBI) did not show high radiotracer uptake. Treatment with cinacalcet was initiated but yielded a poor response, so he finally underwent bilateral neck exploration and total parathyroidectomy without autotransplantation. Histology diagnosis showed MGD due to three hyperplastic parathyroid glands plus one APA (1.7 g weight).

Post-operatively, lab tests showed a nadir PTH of 561 pmol/L and nadir serum-corrected calcium level of 1.75 mmol/L, suggesting post-surgical hypoparathyroidism. Treatment with calcium supplements and calcitriol was initiated, and normal serum levels were recovered.

Considering his young age at diagnosis and the identification of APA and MGD with three hyperplastic parathyroid glands, a screening test of the most likely genetic causes of FHPT was performed. Sequence analysis of DNA obtained from blood identified a heterozygous nonsense variant of *CDKN1B* c.169C>T, p.(Gln57*) (NM 004064.4).

#### Germline genetic analysis and LOH/CNV study in tumour

This *CDKN1B* variant is classified as likely pathogenic based on the established association between the gene, patient’s phenotype, variant’s absence in control populations and type of variant (nonsense). It generates a premature stop codon in exon 1 (of 3 exons) and is predicted to produce a loss of normal protein function, either through protein truncation or nonsense-mediated mRNA decay. This variant was detected in 48% (132 out of 276) NGS reads. As the patient had an aberrant T-cell population, and some pathogenic *CDKN1B* variants have been associated with hematologic malignancy, the identified variant could represent either a germline or somatic variant. Therefore, we performed genetic analyses on other tissues (colon, thyroid, and saliva) [29, 30], and found that all of them showed the presence of this variant; thus, we concluded that c.169C>T was a germline variant. This variant has been reported several times in the Catalogue Of Somatic Mutations In Cancer (COSMIC) in more tissues (skin, breast, prostate, haematopoietic, and lymphoid tissues). These data strongly support the pathogenicity of this variant. This variant is absent in gnomAD and, according to our knowledge, it has not been described as a germline variant in the literature or reported in disease-related variation databases. It should be mentioned that although it is a variant that when expressed somatically is associated with a significant risk phenotype, in this case the patient developed a rather mild tumoral phenotype (PHPT due to APA and three hyperplastic parathyroid glands and aberrant T-cell population without clinical affectation for the moment). The genetic analysis of tumour DNA did not show LOH or CNV.

#### p27/kip1 immunohistochemistry

Immunohistochemical analysis of the excised parathyroid glands (three hyperplastic parathyroid glands and one APA) demonstrated loss of staining for p27 in neoplastic cells. This result is consistent with a lower expression of the tumour suppressor protein due to the described pathogenic variant (Fig. 1b).

#### Clinical evolution after the genetic result

We completed the screening for MEN4-related comorbidities (Table 1). CT scan of the chest and abdomen did not show any abnormalities. Measurement of pancreatic polypeptide (PP) and plasma vasoactive intestinal peptide (VIP) was normal as was a dexamethasone suppression test. Serum CgA (1947.1 μg/L) and serum gastrin (132.6 pmol/L, RV 6.2–54.8) were elevated. Since the proband did not indicate clinical or radiological signs of NETs, these results were attributed to proton pump inhibitor therapy and ESRD [31]. Concerning pituitary hormones, he showed moderated prolactin elevation, hypergonadotropic hypogonadism, and low insulin-like growth factor 1 (IGF1) levels, which could be explained by CKD [32]. Pituitary MRI did not show abnormalities.

#### Family screening after the genetic result

Family members were referred for genetic counselling. In terms of first-degree alive relatives, he had two living sisters and one brother. All of them had undergone predictive testing, which did not detect the *CDKN1B* c.169C>T p.(Gln57*) variant in tested samples (Fig. 2b). Parental samples were not available.

## Case 3

### Medical history and clinical presentation

We present a 44-year-old woman that noticed a neck lump when she was 28. She had no significant medical history. No family history of parathyroid known alterations was reported, but it should be noted that her mother had a history of renal colic episodes. Neck US showed an extrathyroidal hypoechoic nodule of 1.1 cm posterior to the right thyroid lobe and a large cystic nodule (3 cm) on the left side. A neck CT scan revealed the same cystic lesion in a left para-traqueal location, measuring a maximum diameter of 3 cm. It remained unclear whether it was of parathyroid or thyroid origin. Scintigraphy using TcMIBI showed avid tracer uptake near the right thyroid lobe, suggesting a single parathyroid lesion. Fine-needle aspiration biopsy of the cystic nodule showed non-specific cystic content. Lab tests revealed a PHPT pattern with corrected calcium levels of 2.84 mmol/L and PTH of 846 pmol/L. BMD showed normal bone mineralization, and abdominal US excluded renal lithiasis at that moment. Bilateral neck exploration with left cyst excision and right extrathyroidal nodule extraction was performed. The final pathologic diagnosis was MGD due to one parathyroid adenoma (0.1 g weight) and one mainly cystic parathyroid adenoma (3.5 cm diameter, unknown weight due to splitting of the cyst).

After surgery, PTH levels dropped to 409 pmol/L after which she remained asymptomatic and normocalcemic during the post-surgery years. Genetic screening of FHPT forms was performed given the detection of MGD, the existence of a large cystic parathyroid adenoma, and her young age at diagnosis of PHPT. The patient was heterozygous for c.319T>G, p.(Leu107Val) in *CDKN2C* (NM 001262.2).

### Germline genetic analysis and LOH/CNV study in tumour

**Fig. 3:**
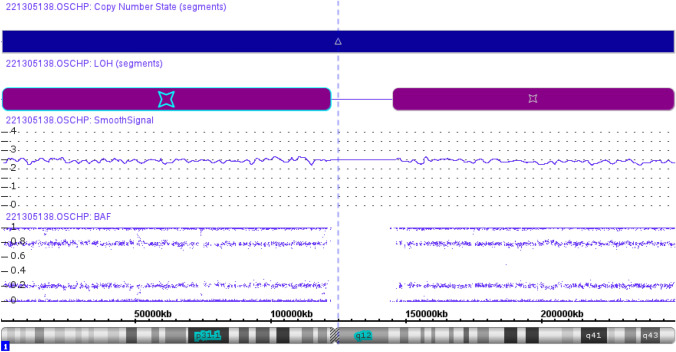
Genome-wide DNA study of loss of heterozygosity (LOH) and copy number variation (CNV) in case 3. CNV track shows a gain of chromosome 1 and B-allele frequency (BAF) track shows uniparental disomy of whole chromosome 1

This *CDKN2C* variant was genetically classified as a variant of uncertain significance (VUS) according to the ACMG criteria. This variant affects a conserved amino acid, and it is predicted to be damaging by most in silico tools. Only two missense variants in *CDKN2C* are currently reported in the HGMD: (1) one in association with MEN1 and (2) the other in association with parathyroid adenoma [16, 33]. Agarwal et al. reported a missense germline variant in a family with parathyroid tumours in which a decrease in protein expression as a probable cause of sporadic endocrine tumours was found [16]. The c.319T>G variant in *CDKN2C* has been reported in gnomAD in 9/251,454 alleles with a low allele frequency (3.58 × 10^−5^). It should be noted that the gnomAD database excludes individuals known to be affected with the severe paediatric disease in addition to their first-degree relatives [34]. The clinical age-related penetrance in patients with *CDKN2C* variants could be low or limited due to the small number of cases described in the literature. Functional studies would help to a better classification of this variant. Sporadic adenomas have not been extensively investigated for candidate variants in *CDKI* genes and specifically for *CDKN2C*. This gene has been examined in a small sporadic adenomas series and to date, has no known identified variant [34, 35]. In this case, the analysis of LOH and CNV in tumour sample showed a gain of whole chromosome 1 and the genotype for three copies were identical leading to somatic uniparental disomy of chromosome 1 (Fig. 3). The detection of this event may reinforce the pathogenicity of this variant although this result should be viewed with caution.

### Clinical evolution after the genetic result

After finding the *CDKN2C* variant, physical, imaging, and lab exams were performed following MEN1 guidelines (Table 1). Concerning dermatological features, physical examination revealed a preauricular right tag and seborrheic keratosis. A CT scan of the abdomen and chest showed uterine leiomyomas and some ovarian cysts. No adrenal alteration was seen. MRI indicated a nodule (0.6 × 0.7 cm), suggestive of cystic non-functional pituitary microadenoma, and the only alteration of pituitary lab test was mild elevated prolactin (1358.64 pmol/L, RV 212.6–1034.5) without clinical significance. Urinary free cortisol level was elevated twice (225.5 pg/24 h and 156.9 pg/24 h; RV 36–137), but no clinical features of Cushing syndrome (CS) were detected. In addition, a salivary cortisol and dexamethasone suppression test showed no alterations, so CS diagnosis was not confirmed. Screening for asymptomatic NETs showed only a non-specific mild elevated CgA (110.3 μg/L).

### Family screening after the genetic result

First-degree relatives underwent genetic counselling. Proband’s mother (II.7, Fig. 2c) carried the variant. She was offered clinical assessment, including biochemical and imaging tests of the pancreas, the pituitary, and the adrenal glands. Lab tests showed hypercalcemia (2.97 mmol/L) and elevated PTH (2423 pmol/L). US of the neck revealed a hypoechoic nodule (0.5 × 0.3 cm) posterior to the left thyroid lobe and scintigraphy using TcMIBI showed an increased tracer uptake in the same location, so she was diagnosed with PHPT. Abdominal US confirmed the presence of renal lithiasis. Due to densitometric osteoporosis (lumbar spine 0.637 g/cm^2^ and *T* score − 4.69 SD; femoral neck 0.593 g/cm^2^ and *T* score − 3.23 SD), alendronate was prescribed. We performed unilateral neck exploration with the removal of a single parathyroid adenoma. PTH levels declined to normal after excision. CT scan of the chest and abdomen did not show abnormalities. Regarding pituitary hormones, lab test showed a low morning serum cortisol level (5.04 µg/dL; RV 6.02–18.4) with low adrenocorticotropin hormone level (4.2 pg/mL; RV 7.2–63.3), pattern suggesting secondary adrenal insufficiency. We performed a 250 µg cosyntropin stimulation test, which showed normal post-stimulation cortisol levels: (1) 30 min − 26.9 µg/dL and (2) 60 min 31.9 µg/dL (RV > 14 µg/dL). No other lab pituitary hormone alterations or radiological abnormalities of the gland were detected.

Other family members (proband’s father, sister, grandmother, and great uncles) did not present the variant and showed normal phospho-calcium metabolism lab tests (Fig. 2c).

## Discussion

We report three novel *CDKIs* germline variants in three different patients with PHPT identified during the course of screening for FHPT causes: (1) two likely pathogenic variants of *CDKN1B* gene (cases 1 and 2) and (2) one VUS of *CDKN2C* gene (case 3).

As stated above, *CDKIs* germline mutations have been proposed as a cause of MEN1-like syndromes without *MEN1* mutation. This situation is called MEN4 when a *CDKN1B* germline mutation is found. However, the clinical course of patients affected by mutations in *CKDIs* is still poorly understood since few cases have been reported until now. We reviewed medical literature and genetic databases (ClinVar, HGMD) and found that a few more than 100 cases with MEN4, including 52 different variants of *CDKN1B*, have been reported to date [12, 16, 20, 29, 36–54]. As supplementary material, we added a table with the total number of *CDKIs* pathogenic/likely pathogenic/VUS germline variants thus far reported along with the type of parathyroid lesion or other tumours found (if reported) and pathogenicity level of the variants, which was assessed following the ACMG guidelines [26] (see supplementary material Appendix 1). It should be noted that 34 of these *CDKN1B* variants have been reported from a single submitter as criteria provided and 10/55 are classified as VUS. All these facts support that so very little knowledge about the clinical implications of *CDKIs* pathogenic variants is available to date. In addition, four other variants of *CDKN1B* have been reported in patients with medulloblastoma, paraganglioma, acute lymphoblastic leukaemia and familial colorectal cancer [30, 55–57].

Germline mutations in three other *CDKI* genes (*CDKN2B*, *CDKN2C*, and *CDKN1A*) were found in MEN1-like cases without detectable *MEN1* mutations [16]. Afterall, a reliable prevalence of *CDKIs* mutations in MEN1-like patients or patients with PHPT is difficult to estimate as genetic screening of these genes has not been widely established. We report three novel *CDKIs* germline mutations in three different patients with PHPT. The possibility of including these cases within the term MEN4 or MEN1-like states should be assessed.

MEN4–PHPT affects approximately 80–90% of the reported MEN4 cases to date [7, 53]. This finding is in accordance with MEN1 classical presentation [58]. On the contrary, in most MEN4 cases, the clinical course of PHPT seems to be less severe than in patients with MEN1 syndrome and exhibits a female predominance. Only one case of MEN4 presented recurrent PHPT after subtotal parathyroidectomy [39, 53]. All cases that we reported agree with this as all of them showed remission of PHPT after surgery to date (at least nine years follow-up). However, moderate to severe hypercalcemia was seen in our three cases in contrast to other cases of PHPT associated with *CDKIs* mutations [53] (Table 1). Considering that most of these patients presented remission after surgery, differing from many MEN1 cases, a minimally invasive parathyroid surgery could be contemplated. Regarding endocrine histology, most of the cases of PHPT-related to *CDKIs* mutations described to date were caused by a single parathyroid adenoma [53], except for a few cases of MGD [16, 39]. To our knowledge, we report for the first time two cases of APA and one case of cystic parathyroid adenoma related to *CDKIs* germline mutations (Table 1).

Regarding pituitary neoplasms, up to now, some cases of these tumours were described in patients with MEN4 (43%) [12, 38, 45, 47, 49, 50, 52, 53]. These findings agree with the estimated prevalence of pituitary tumours in patients with MEN1 (40%). Recently, in a large cohort of 211 patients (mostly paediatric) with Cushing disease, five germline *CDKN1B* variants were identified (2.6%) [50]. In addition, three germline *CDKN1B* variants were detected in three paediatric patients with pituitary adenomas, with no other manifestations related to MEN4 [47]. In addition, one case of macroprolactinoma was described in a case with germline *CDKN1A* mutation [16]. Somatotroph, corticotroph, and non-functioning pituitary adenomas have been found to be the most frequent pituitary tumours in MEN4 cases. On the contrary, prolactinomas seem less frequent in MEN4 patients [45, 52]. Non-functioning microadenomas are usual in the general population, and it remains challenging to distinguish between incidentalomas and MEN-related pituitary tumours [53]. In this regard, a non-functioning microadenoma was detected in case 3. The age of onset of pituitary tumours in MEN4 is still unclear due to the few number of cases reported until now.

Concerning NETs, we did not find any radiological image or analytical alteration suggestive of tumour in our cases. That finding fits with the last data about MEN4 cases, which indicates a lower prevalence of NETs than in MEN1 [53].

Other tumours, such as meningiomas, liver haemangiomas, skin tumours, adrenal nodes, colon cancer, breast cancer, and genitourinary neoplasms, have been described in patients with germline mutations of *CDKIs* [12, 16, 20, 36, 38, 53]. We describe a colon adenoma with low-grade dysplasia and atypical lipomas in case 1 and a colon neoplasia in her mother who carried the same variant in *CDKN1B*. In addition, we report a malignant hemopathy (aberrant T-cell population) in a *CDKN1B* carrier (case 2).

In the firsts two cases (cases 1 and 2), genetic analysis of tumour samples showed nor LOH neither CNV for *CDKN1B*, findings that agree with those in the literature. We reviewed recent data of MEN4 published cases describing *CDKN1B* pathogenic variants that have analysed tumour tissue to assess the presence of a possible LOH. In most of the cases, tumour testing failed to demonstrate a second somatic event in the wildtype allele. Therefore, in contrast to MEN1 syndrome, haploinsufficiency for *CDKN1B* appears to be enough for tumours to occur in MEN4 syndrome [41, 53, 59]. Conversely, in case 3, genetic tumour testing showed uniparental disomy of chromosome 1, including *CDKN2C* gene, which could support a pathogenic role of this variant. Another germline variant of *CDKN2C* with LOH was described in a patient with parathyroid adenoma [33]. In addition, some reports propose the contribution of *CDKN2C* as tumour suppressor gene in different neoplasms [60–63]. However, further studies are required to establish whether *CDKN2C* germline pathogenic variants cause genetic predisposition to FHPT or multiple endocrine neoplasms. In addition, immunohistochemistry testing for p18 (not available in our laboratory) could be of interest in future studies of similar cases to determine the protein expression in tumour tissue.

To summarize, we report two novel likely pathogenic variants of *CDKN1B* gene in patients with suspected familial PHPT. The first one was found in a patient with atypical parathyroid adenoma, atypical lipomas, and a colon tubule-villous adenoma with low-grade dysplasia. His mother, who carried the same variant, died from colon neoplasia and once presented with non-studied hypercalcemia. The second one found in a patient with PHPT is associated with aberrant T-cell population and other comorbidities, such as obesity, subclinical hypothyroidism, and chronic idiopathic axonal polyneuropathy. In addition, we describe a novel variant in *CDKN2C* gene with uniparental disomy in tumour sample that was classified as uncertain significance in a patient with PHPT and a non-functional pituitary adenoma. Although the case 3 variant was classified as VUS, we would like to highlight its clinical relevance since both the proband and her mother, who carried the same variant, presented PHPT.

As a reflection, we would like to point out that genetic screening of FHPT could be considered in patients with features such as APA or in the presence of other tumours apart from PHPT. Considering all reported cases of MEN1-like patients with *CDKIs* germline mutations with PHPT as the predominant feature, we theorize that patients with mutations in different *CDKIs* apart from *CDKN1B* (such as *CDKN2C*) may be encompassed in the nomenclature of MEN4. However, further studies should be done for a better understanding of this entity, and development of specific guidelines for these patients should be established. New germline variants described in this study can be added to the current knowledge. In conclusion, we can say that germline mutations in *CDKIs* may represent an etiology of FHPT. Therefore, these genes should be included in gene panels for screening this disease.

## Supplementary Information

Below is the link to the electronic supplementary material.

Supplementary file1 (XLSX 17 KB)
